# The *Ralstonia solanacearum* csp22 peptide, but not flagellin‐derived peptides, is perceived by plants from the *Solanaceae* family

**DOI:** 10.1111/pbi.12874

**Published:** 2018-01-22

**Authors:** Yali Wei, Carlos Caceres‐Moreno, Tamara Jimenez‐Gongora, Keke Wang, Yuying Sang, Rosa Lozano‐Duran, Alberto P. Macho

**Affiliations:** ^1^ Shanghai Center for Plant Stress Biology CAS Center for Excellence in Molecular Plant Sciences Shanghai Institutes of Biological Sciences Chinese Academy of Sciences Shanghai China; ^2^ University of Chinese Academy of Sciences Beijing China

**Keywords:** ralstonia, elicitor, flagellin, flg22, csp22, bacterial wilt

## Abstract

*Ralstonia solanacearum*, the causal agent of bacterial wilt disease, is considered one of the most destructive bacterial pathogens due to its lethality, unusually wide host range, persistence and broad geographical distribution. In spite of the extensive research on plant immunity over the last years, the perception of molecular patterns from *R. solanacearum* that activate immunity in plants is still poorly understood, which hinders the development of strategies to generate resistance against bacterial wilt disease. The perception of a conserved peptide of bacterial flagellin, flg22, is regarded as paradigm of plant perception of invading bacteria; however, no elicitor activity has been detected for *R. solanacearum* flg22. Recent reports have shown that other epitopes from flagellin are able to elicit immune responses in specific species from the *Solanaceae* family, yet our results show that these plants do not perceive any epitope from *R. solanacearum* flagellin. Searching for elicitor peptides from *R. solanacearum*, we found several protein sequences similar to the consensus of the elicitor peptide csp22, reported to elicit immunity in specific *Solanaceae* plants. A *R. solanacearum* csp22 peptide (csp22^Rsol^) was indeed able to trigger immune responses in *Nicotiana benthamiana* and tomato, but not in *Arabidopsis thaliana*. Additionally, csp22^Rsol^ treatment conferred increased resistance to *R. solanacearum* in tomato. Transgenic *A. thaliana* plants expressing the tomato csp22 receptor (SlCORE) gained the ability to respond to csp22^Rsol^ and became more resistant to *R. solanacearum* infection. Our results shed light on the mechanisms for perception of *R. solanacearum* by plants, paving the way for improving current approaches to generate resistance against *R. solanacearum*.

## Introduction

*Ralstonia solanacearum* is a soil‐borne bacterial pathogen able to cause disease in more than 250 plant species (Jiang *et al*., 2017; Mansfield *et al*., 2012). It has an extremely versatile lifestyle, surviving in water, soil and plant debris. *R. solanacearum* perceives root exudates and employs its flagellum for movement and efficient root invasion (Tans‐Kersten *et al*., 2001; Yao and Allen, 2006). After invading plant tissues through wounds, root tips or cracks at the sites of lateral root emergence, *R. solanacearum* colonizes the root cortex, reaches the vasculature and spreads through xylem vessels, colonizing the plant systemically (Mansfield *et al*., 2012). Subsequent massive bacterial replication leads to a quorum sensing‐dependent metabolic switch in bacterial cells (Khokhani *et al*., 2017; Perrier *et al*., 2016; Peyraud *et al*., 2016), triggering the production of virulence factors and exopolysaccharide (EPS; McGarvey *et al*., 1999), which causes the disruption of the plant vascular system and eventual plant wilting (Digonnet *et al*., 2012; Turner *et al*., 2009). Given its lethality, unusually wide host range, persistence and broad geographical distribution, *R. solanacearum* is currently considered one of the most destructive bacterial pathogens in agricultural systems.

To fend off pathogens, plants have evolved to perceive different microbial molecules. Conserved pathogen‐associated molecular patterns (PAMPs) can be perceived at the plant cell surface by receptors localized at the plasma membrane, termed pattern‐recognition receptors (PRRs (Zipfel, 2014)). Moreover, proteins injected by pathogens inside plant cells can also be perceived, in a direct or indirect manner, by intracellular receptors containing nucleotide‐binding and leucine‐rich repeat domains (NLRs; Khan *et al*., 2016). Applying a broad view of the plant immune system, bacterial elicitors perceived either extracellularly and intracellularly can be generally regarded as invasion patterns (Cook *et al*., 2015), and their perception leads to the activation of immune responses aimed at restricting bacterial infection (Bigeard *et al*., 2015; Boller and Felix, 2009; Tsuda and Katagiri, 2010). Despite the extensive research on plant immunity over the last years, little is known about the perception of immune elicitors from *R. solanacearum*, and our understanding of *R. solanacearum* perception by plants comprises almost exclusively the recognition of effector proteins delivered inside plant cells through the type III secretion system (Deslandes and Genin, 2014; Huet, 2014; Jayaraman *et al*., 2016). As a consequence, current agricultural approaches to control bacterial wilt disease caused by *R. solanacearum* are limited (Huet, 2014; Jiang *et al*., 2017), suggesting a need for identification of additional immune elicitors present in *R. solanacearum* that could be perceived by different plant species, and a characterization of the plant immune components that mediate this perception. Currently, different species from the *Solanaceae* family show varying degree of resistance to *R. solanacearum*, mostly based on the recognition of specific bacterial type III effectors (Huet, 2014; Poueymiro *et al*., 2009). Similarly, specific tomato cultivars also display some degree of disease resistance dependent on the putative recognition of bacterial exopolysaccharide, which otherwise acts as a virulence factor (Milling *et al*., 2011).

The bacterial flagellum is formed by polymerized flagellin protein (encoded by the FliC gene). The perception of a conserved peptide of bacterial flagellin, flg22, is possibly the best studied example of plant recognition of invading bacteria, and occurs in plants harbouring the PRR FLS2 (Felix *et al*., 1999; Gómez‐Gómez and Boller, 2000). However, no elicitor activity has been detected so far for *R. solanacearum* flg22 (flg22^Rsol^) in Arabidopsis, tomato or tobacco plants (Mueller *et al*., 2012a; Pfund *et al*., 2004), probably due to the presence of several polymorphisms in the amino acid sequence of this peptide compared to flg22 peptides from other bacteria (Mueller *et al*., 2012a; Sun *et al*., 2013). This observation has originated the general assumption that *R. solanacearum* has evolved to evade plant recognition of its flagellin by altering this flg22 sequence.

In recent years, it has been reported that specific plants have evolved to perceive peptides within bacterial flagellin other than flg22 (Cai *et al*., 2011; Clarke *et al*., 2013; Katsuragi *et al*., 2015). Several plants from the *Solanaceae* family are able to perceive a distinct 28 amino acid peptide within flagellin from several *Pseudomonas syringae* strains, named flgII‐28 (Cai *et al*., 2011; Clarke *et al*., 2013). This perception is mediated by the PRR FLS3 (Hind *et al*., 2016) and is restricted to specific species within the *Solanaceae* family, including tomato, potato and pepper (Clarke *et al*., 2013). Other *Solanaceae* plants, such as tobacco, eggplant or *Nicotiana benthamiana*, or non‐*Solanaceae* plants, including Arabidopsis, cannot perceive flgII‐28 (Clarke *et al*., 2013). Whether FLS3 can recognize flgII‐28 from *R. solanacearum* is currently unknown. In the light of these reports, and considering the current limitations in our understanding of plant perception of *R. solanacearum* immune elicitors, we designed a systematic approach with the aim of determining whether other peptides from *R. solanacearum* flagellin could be perceived by plants from the *Solanaceae* family. This work was based on the following reasoning:

1. Tomato is a host for *P. syringae* species harbouring immunogenic flgII‐28. Similarly, specific *Solanaceae* plants may have co‐evolved with *R. solanacearum* and developed specific mechanisms for the perception of *R. solanacearum* flgII‐28 (flgII‐28^Rsol^) or other conserved elicitors.
2. There is no report on the ability of tomato plants (or other non‐*Nicotiana* solanaceous plants) to perceive flgII‐28^Rsol^ or other peptides of *R. solanacearum* flagellin. It is known that *R. solanacearum* flagellin is not an elicitor in Arabidopsis and tobacco (Pfund *et al*., 2004), but these species do not respond to flgII‐28 (Clarke *et al*., 2013). Therefore, a potential elicitor activity of flgII‐28^Rsol^ may have been overlooked in previous studies.
3. There is an example of another bacterial species, *P. cannabina pv. alisalensis* ES4326 (*Pcal* ES4326; formerly known as *P.s. maculicola*), whose flgII‐28, but not flg22, is recognized by tomato (Clarke *et al*., 2013; Hind *et al*., 2016).
4. Most elicitation assays published to date have been performed in leaf tissue or cultured cells, without taking into consideration root responses. Given that root is the most common entry point into plant tissues for *R. solanacearum,* the ability of this organ to perceive and respond to invading bacteria is of special relevance.

In this work, we tested a synthetic flgII‐28^Rsol^ peptide and full *R. solanacearum* flagellin for elicitation in different plant species, including tomato, pepper, eggplant, tobacco and *N. benthamiana*, using either leaf or root tissues. Our results show that none of these species is able to respond to *R. solanacearum* flagellin.

We next sought to identify other *R. solanacearum* elicitor peptides that could be perceived by plants. The csp22 peptide is present in the conserved COLD‐SHOCK PROTEIN from several bacterial species (Felix and Boller, 2003) and is perceived by PRRs from several *Solanaceae* species (Saur *et al*., 2016; Wang *et al*., 2016). Our results show that specific *Solanaceae* species, including tomato, tobacco and *N. benthamiana*, can perceive the csp22 peptide derived from *R. solanacearum* cold‐shock protein (csp22^Rsol^), and that the reported csp22 receptor CORE (Wang *et al*., 2016) confers responsiveness to csp22^Rsol^ in Arabidopsis. Moreover, csp22^Rsol^ induces immune responses that are able to restrict *R. solanacearum* growth in tomato. This work allowed us to uncover a *R. solanacearum* peptide perceived by a PRR present in specific *Solanaceae* species, paving the way for the potential design of novel strategies to confer or increase resistance to *R. solanacearum* in additional plant species through interfamily transfer of the associated PRR.

## Results

### *Solanaceae* plants cannot perceive flg22^Rsol^ or flgII‐28^Rsol^ in leaves or roots

**Figure 1:**
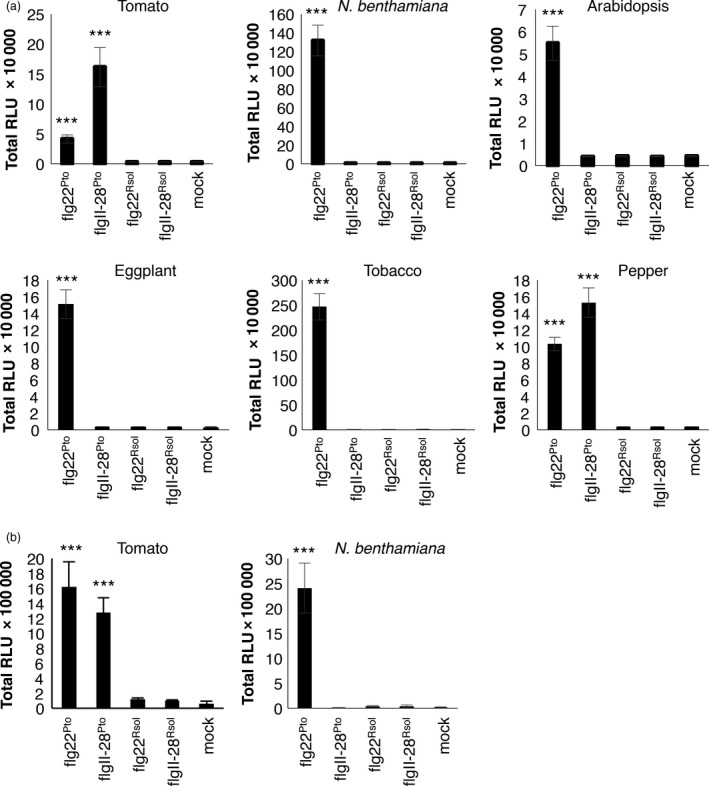
Flg22 or flgII‐28 from *Ralstonia solanacearum* does not elicit responses in *Solanaceae* plants. Oxidative burst triggered by 100 nm of the indicated peptides or water (mock) in leaves (a) or roots (b) of plants from the indicated species, measured in a luminol‐ or L‐012‐based assay, respectively, as relative luminescence units (RLU) during 60 min. Values are average ± SE (*n* = 8). Asterisks indicate significant differences compared to the mock control according to a Student's *t*‐test (*P* < 0.001). The experiments were repeated at least three times with similar results.

The amino acid sequence of the FliC gene product in *R. solanacearum* GMI1000 contains both flg22 and flgII‐28 sequences, showing several polymorphisms compared to flg22 and flgII‐28 from *P. syringae* strains (Clarke *et al*., 2013; Mueller *et al*., 2012a; Figure S1). To test whether flgII‐28^Rsol^ could act as an elicitor of immune responses in *Solanaceae* plants, we monitored the elicitor‐triggered burst of reactive oxygen species (ROS) in leaf tissues, which is a widely used readout to test elicitor activities in different plant species (Clarke *et al*., 2013; Hind *et al*., 2016). Our results show that none of the plant species tested (tomato, *N. benthamiana*, eggplant, tobacco, pepper, and Arabidopsis, the latter used as control) showed a detectable response to flgII‐28^Rsol^ (Figures 1a and S2). As previously reported, all these species responded to flg22 from *P. syringae pv tomato* DC3000 (flg22^Pto^), while none of them responded to flg22^Rsol^ (Figures 1a and S2). As reported before, only tomato and pepper responded to flgII‐28 from *P. syringae pv tomato* T1 (flgII‐28^Pto^) (Figures 1a and S2; Clarke *et al*., 2013).

As a soil‐borne pathogen, *R. solanacearum* mostly invades plant tissues through the roots. As most of the elicitation tests commonly used in plants are based on leaf tissue (or cultured cells), we sought to determine whether roots of tomato or *N. benthamiana* plants could respond to flg22^Rsol^ or flgII‐28^Rsol^, setting up an assay to measure ROS in roots of these plant species. However, elicitation patterns in roots mirrored those in leaves for these species and peptides: both species responded to flg22^Pto^, only tomato responded to flgII‐28^Pto^, and none of them responded to flg22^Rsol^ and flgII‐28^Rsol^ (Figures 1b and S3).

As an alternative method to test activation of PRR‐dependent responses, we used a phosphorylation‐dependent specific antibody to determine the phosphorylation of mitogen‐activated protein kinases (MAPKs), which is a well‐established elicitor‐triggered response. The observed patterns of peptide‐induced activation of immunity, measured as MAPK activation in tomato, *N. benthamiana,* or Arabidopsis seedlings resembled those observed in ROS assays, with none of them responding to the flagellin‐derived peptides from *R. solanacearum* (Figure S4).

### *Solanaceae* plants cannot perceive other peptides from *Ralstonia solanacearum* flagellin in leaves or roots

**Figure 2:**
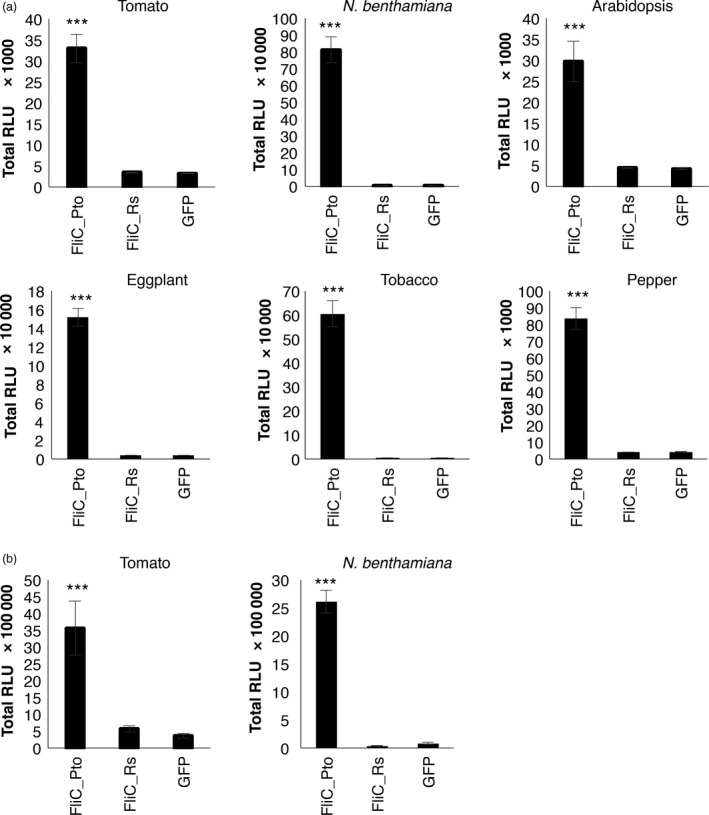
Purified recombinant flagellin from *Ralstonia solanacearum* does not elicit responses *Solanaceae* plants. Oxidative burst triggered by 100 nm of the indicated recombinant proteins in leaves (a) or roots (b) of plants from the indicated species, measured in a luminol‐ or L‐012‐based assay, respectively, as relative luminescence units (RLU) during 60 min. Values are average ± SE (*n* = 8). Asterisks indicate significant differences compared to the GFP control according to a Student's *t*‐test (*P* < 0.001). The experiments were repeated at least three times with similar results.

To investigate whether *Solanaceae* plants can perceive other peptides from *R. solanacearum* flagellin different from flg22 and flgII‐28, we purified the full‐length flagellin protein (FliC) from *R. solanacearum* (FliC_Rs), fused to an N‐terminal 6His‐MSB tag and a C‐terminal Strep tag (His‐MSB‐FliC_Rs‐Strep; Figure S5). FliC from *P. syringae pv tomato* (His‐MSB‐FiC_Pto‐Strep) was used as a positive control, and a GFP recombinant protein (His‐MSB‐GFP‐Strep) as negative control. All proteins were expressed in *Escherichia coli* and purified as described in the methods section. The recombinant purified GFP did not trigger a detectable response in leaves of any of the plants tested (Figure 2a), indicating that our purified recombinant proteins do not contain significant levels of contaminants from *E. coli* with eliciting activity, such as EF‐Tu. All the plant species tested (tomato, *N. benthamiana*, eggplant, tobacco, pepper, and Arabidopsis) responded to His‐MSB‐FiC_Pto‐Strep, while none of them showed a detectable response to His‐MSB‐FliC_Rs‐Strep (Figures 2a, S6). The same pattern was observed in roots of tomato and *N. benthamiana* (Figures 2b, S7). To rule out a specific effect of the His‐MSB‐Strep tag or the purification process on the absence of elicitation by *R. solanacearum* flagellin, we expressed our recombinant proteins fused to a N‐terminal 6His‐maltose‐binding protein (His‐MBP; Figure S8a) tag in *E. coli* and purified them as described in the methods section (Figure S8b and c). Further analysis using liquid chromatography followed by tandem mass spectrometry (LC‐MS/MS) confirmed that our recombinant protein contains the full sequence of *R. solanacearum* FliC (Figure S8d). However, recombinant His‐MBP‐FliC_Rs did not elicit a detectable response in tomato, *N. benthamiana*, or Arabidopsis (Figure S9). Of note, during the set‐up of our assays, we noticed that gel filtration column buffer containing DTT caused a detectable luminescent signal in ROS assays (data not shown), and therefore, our recombinant proteins were eluted in buffer without DTT.

Our experiments indicate that none of the tested plant species are able to perceive flg22^Rsol^, flgII‐28^Rsol^ or full recombinant flagellin from *R. solanacearum*.

### Specific *Solanaceae* plants can perceive the csp22 peptide from *Ralstonia solanacearum*

**Figure 3:**
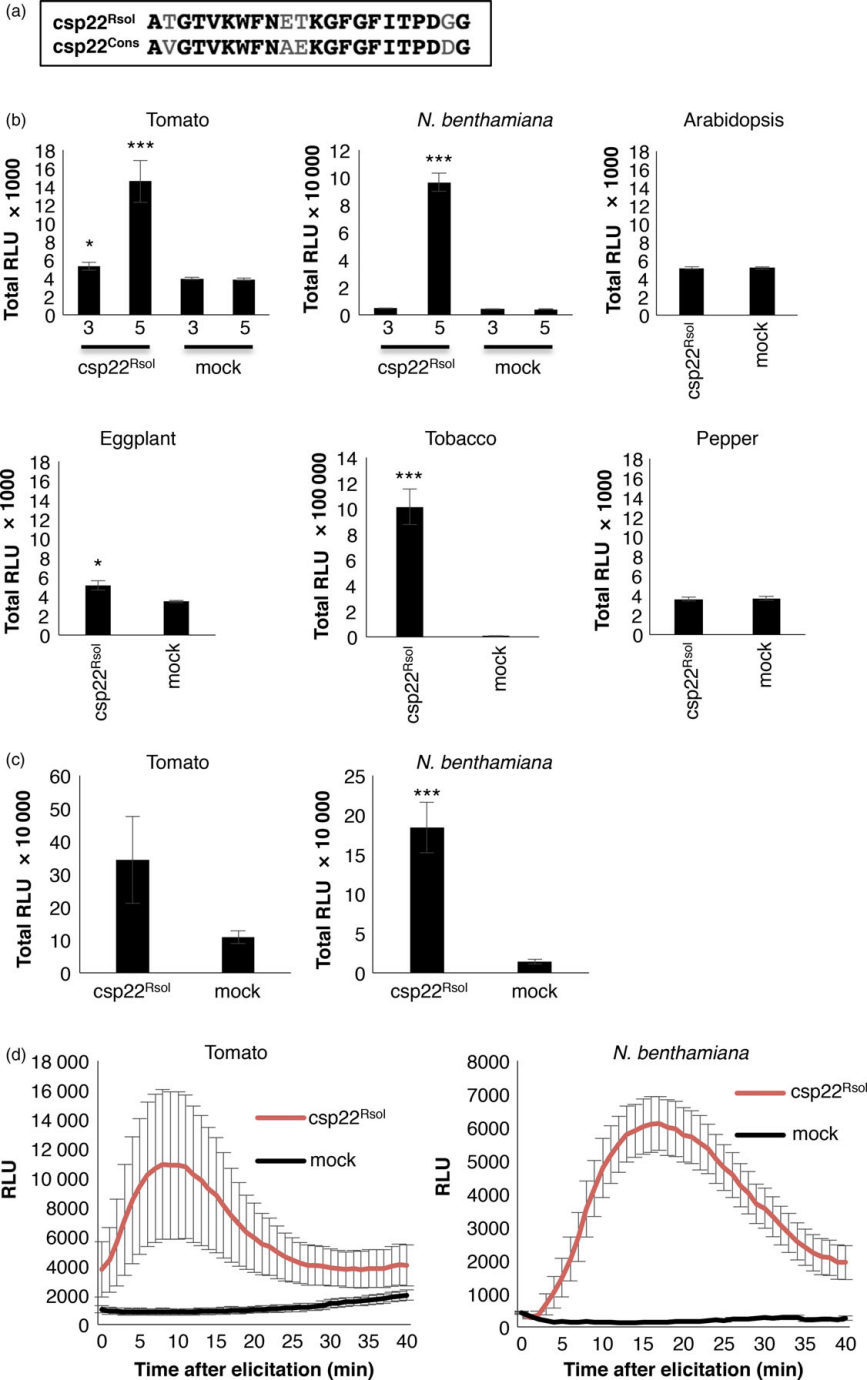
Csp22 from *Ralstonia solanacearum* is perceived in specific *Solanaceae* plants. (a) Sequence alignment of the csp22 sequences used in this study. csp22 peptide (csp22^Rsol^) corresponds to the csp22 sequence from *R. solanacearum*; csp22^Cons^ corresponds to the consensus csp22 sequence used in the original publication by Felix and Boller (2003). (b and c) Oxidative burst triggered by 100 nm of csp22^Rsol^ or water (mock) in leaves (b) or roots (c) of plants from the indicated species, measured in a luminol‐ or L‐012‐based assay, respectively, as relative luminescence units (RLU) during 60 min. Numbers 3 and 5 at the *x*‐axis indicate the age of the plants: 3‐ and 5 weeks old, respectively. Arabidopsis, eggplant, tobacco and pepper plants used in (b) were 5 weeks old. Seedlings used in (c) were 7 days old (tomato) and 11 days old (*Nicotiana benthamiana*). (d) Dynamics of the early reactive oxygen species (ROS) burst from the assays described in (c). Values are average ± SE (*n* = 8). Asterisks indicate significant differences compared to the mock control according to a Student's *t*‐test (*P* < 0.001). The experiments were repeated at least three times with similar results.

**Figure 4:**
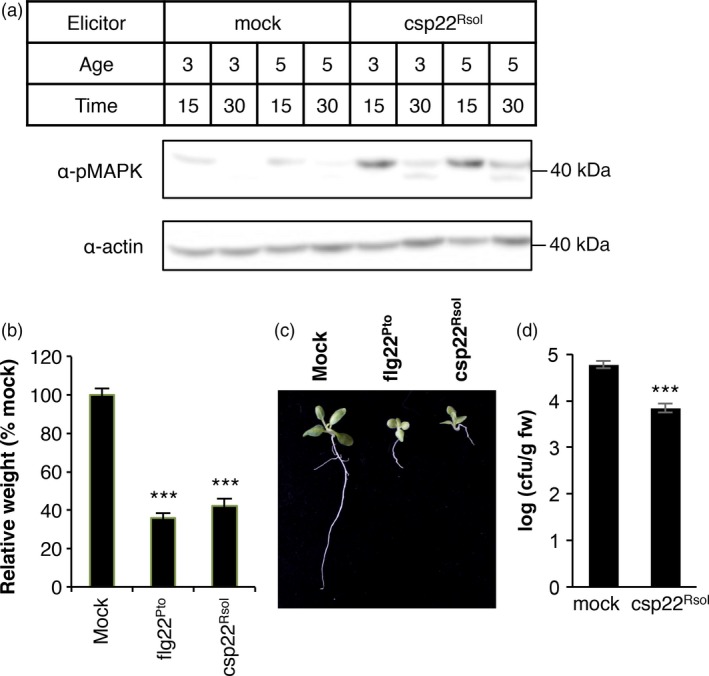
Csp22 from *Ralstonia solanacearum* triggers additional early and late PRR‐dependent immune responses in *Solanaceae* plants. (a) MAPK activation assay in leaves of 3‐ or 5‐week‐old *Nicotiana benthamiana* plants after treatment with 1 μm csp22^Rsol^ or water (mock) for 15 or 30 min as indicated. Immunoblots were analysed using antiphosphorylated MAPK antibody (α‐pMAPK). Immunoblots were also analysed using α‐actin antibody to verify protein accumulation. Molecular weight (kDa) marker bands are indicated for reference. (b) Growth inhibition of *N. benthamiana* seedlings treated with 100 nm flg22^Pto^, csp22^Rsol^ or water (mock) for 9 days. Values represent seedling weigh shown as percentage of mock‐treated seedlings (average ± SE;* n* = 12). (c) Photograph of seedlings after exposure to the different elicitors in (b). (d) Growth of *R. solanacearum* GMI1000 in tomato (cultivar M82) leaves pretreated with 1 μm csp22^Rsol^ or water (mock) for 24 h and then syringe‐infiltrated with a 10^6^ cfu/mL bacterial inoculum. Bacterial growth was determined 3 days after inoculation. Values are mean ± SE (*n* =* *3). Asterisks indicate significant differences compared to the mock control according to a Student's *t*‐test (*P* < 0.001). All experiments were performed three times with similar results.

In a search for further *R. solanacearum* elicitors that could be perceived by PRRs in *Solanaceae* plants, we noticed that *R. solanacearum* encodes at least four proteins with peptides similar to the csp22 consensus peptide from the bacterial COLD‐SHOCK PROTEIN (CSP; originally isolated from *Staphylococcus aureus*; Figure S10), which elicits responses in *Solanaceae* plants (Felix and Boller, 2003). The csp22‐like peptide from the *R. solanacearum* GMI1000 protein CspD3 (encoded by the RSp0002 locus) is similar to the csp22 consensus peptide, showing four amino acid polymorphisms (Figures 3a, S10), and it was therefore named csp22^Rsol^ and used for further analysis. To determine if the csp22^Rsol^ is able to elicit immunity, we performed ROS assays with purified csp22^Rsol^ in leaves of *Solanaceae* plants from different species. Our results show that csp22^Rsol^ triggers a detectable ROS burst in tomato, *N. benthamiana*, and tobacco leaves, while the elicitation in eggplant was barely detectable (Figures 3b, S11). On the contrary, csp22^Rsol^ did not trigger a detectable response in pepper (Figures 3b, S11). The response to csp22 in *N. benthamiana* has been reported to show an age‐dependent pattern, being stronger in older plants (Saur *et al*., 2016; Wang *et al*., 2016). Similarly, our results show that leaves from 5‐week‐old tomato and *N. benthamiana* plants showed a stronger and more reproducible response to csp22^Rsol^ compared to those from 3‐week‐old plants (Figures 3b, S11). Interestingly, although the response in leaves is age dependent, roots of tomato and *N. benthamiana* seedlings (7‐ and 11 days old, respectively) consistently responded to csp22^Rsol^ (Figure 3c and d). We also found that csp22^Rsol^ is able to elicit the activation of MAPKs in *N. benthamiana* leaves (Figure 4a), and this response was occasionally stronger and more sustained in 5‐week‐old plants compared to 3‐week‐old plants (data not shown).

Both ROS and MAPK activation take place within minutes after the perception of a bacterial elicitor by plant PRRs (Bigeard *et al*., 2015; Boller and Felix, 2009). The activation of PRR‐triggered immunity in Arabidopsis seedlings is often associated with an inhibition of growth, which becomes evident after sustained exposure to an immune elicitor (Gómez‐Gómez *et al*., 1999). To determine whether csp22^Rsol^ is also able to trigger late immune‐associated responses in *Solanaceae* plants, we set up a seedling growth inhibition assay for *N. benthamiana* seedlings. The results show that sustained exposure to csp22^Rsol^ caused an inhibition of growth in *N. benthamiana* seedlings, comparable to that caused by flg22^Pto^ (Figure 4b). This effect was especially dramatic in the root of seedlings exposed to csp22^Rsol^ (Figure 4c). Altogether, our results indicate that csp22^Rsol^ triggers early and late PRR‐dependent immune responses in specific *Solanaceae* plants in both roots and aerial parts.

### The perception of csp22^Rsol^ leads to disease resistance to *Ralstonia solanacearum* in tomato plants

The effective activation of PRR‐dependent immune responses leads to the strengthening of plant cells and protection against subsequent bacterial infections (Bigeard *et al*., 2015; Boller and Felix, 2009). To determine whether perception of csp22^Rsol^ can trigger effective disease resistance in tomato plants, we inoculated *R. solanacearum* in tomato leaves 1 day after treatment with csp22^Rsol^ or water (as mock control). Pretreatment with csp22^Rsol^ was able to protect tomato leaves against a subsequent infection by *R. solanacearum*, as reflected by the significant reduction in bacterial growth in csp22^Rsol^ compared to mock‐treated leaves (Figure 4d). This result indicates that csp22^Rsol^ is able to trigger the activation of effective immune responses against *R. solanacearum* in tomato cells.

### The tomato SlCORE receptor confers responsiveness to csp22^Rsol^

**Figure 5:**
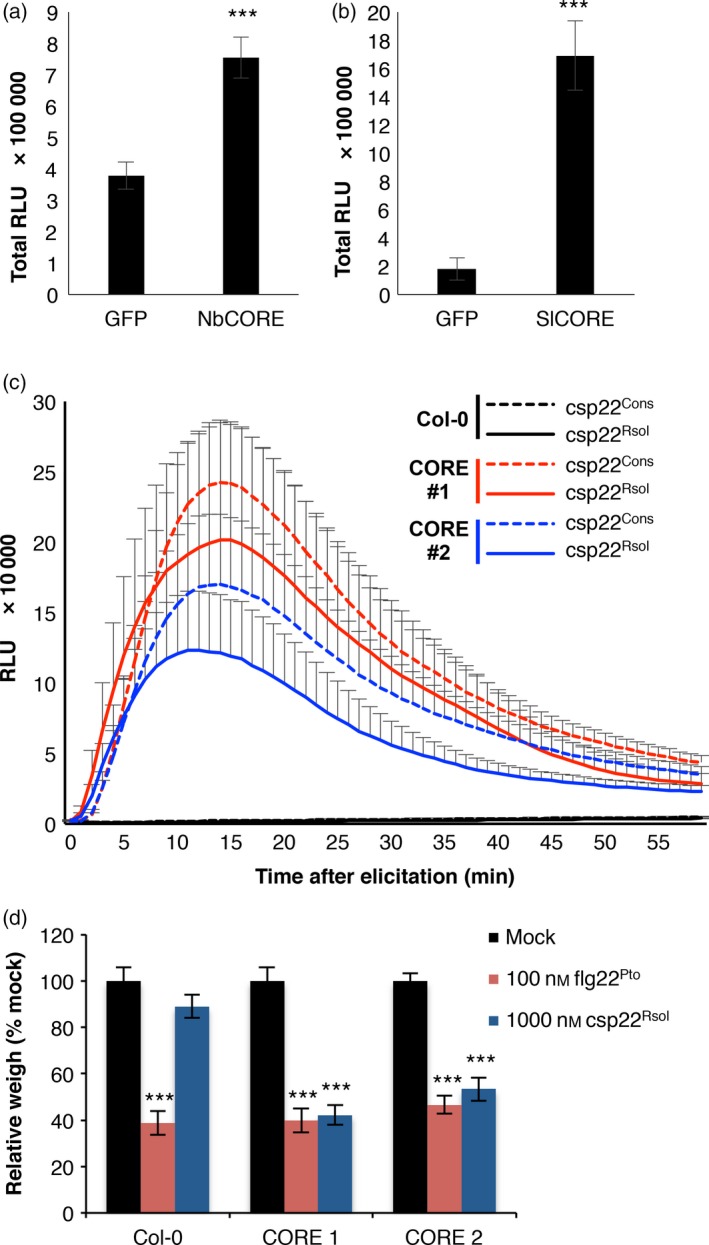
The SlCORE receptor confers responsiveness to csp22 from *Ralstonia solanacearum*. (a and b) Oxidative burst triggered by 100 nm of csp22^Rsol^ in 5‐week‐old *Nicotiana benthamiana* leaves expressing GFP (control), NbCORE (a) or SlCORE (b), measured in a L‐012 (luminol)‐based assay as relative luminescence units (RLU) during 60 min. (c) Dynamics of oxidative burst triggered by 1 μm csp22 consensus peptide (csp22^Cons^) or csp22^Rsol^ in leaves of Col‐0 wt Arabidopsis plants, or two independent Arabidopsis transgenic lines expressing SlCORE, measured in a luminol‐based assay as relative luminescence units (RLU) during 50 min. Values are average ± SE (*n* = 8). (d) Growth inhibition of seedlings of the Arabidopsis lines mentioned in (c) treated with 100 nm flg22^Pto^, 1 μm csp22^Rsol^ or water (mock) for 9 days. Values represent seedling weigh shown as percentage of mock‐treated seedlings (average ± SE;* n* = 12). Asterisks indicate significant differences compared to the control according to a Student's *t*‐test (*P* < 0.001). All experiments were performed three times with similar results.

Several *Solanaceae* plants can perceive csp22, and this perception has been recently associated to specific PRRs in *N. benthamiana* and tomato (Saur *et al*., 2016; Wang *et al*., 2016). The *N. benthamiana* NbCSPR has been reported to confer responsiveness to csp22, but the presence of a tomato sequence homolog for this PRR is unclear (Saur *et al*., 2016). Another report associates this responsiveness in *N. benthamiana* and tomato to the presence of a different PRR, named NbCORE or SlCORE, respectively (Wang *et al*., 2016). As we can detect a clear response to csp22^Rsol^ in both *N. benthamiana* and tomato, we tested whether NbCORE and SlCORE are associated with the perception of csp22^Rsol^. To this end, we first used *Agrobacterium tumefaciens* to transiently overexpress NbCORE or SlCORE in 4‐week‐old *N. benthamiana* leaves. Control leaves overexpressing green‐fluorescent protein (GFP) showed weak and nonconsistent response to csp22^Rsol^, in keeping with our previous results using young *N. benthamiana* leaves (Figures 3b, S11). However, overexpression of NbCORE and, specially, SlCORE led to an enhanced and robust responsiveness to csp22^Rsol^ (Figures 5a–b, S12), suggesting that NbCORE/SlCORE contributes to the plant response against csp22^Rsol^.

Arabidopsis does not perceive the consensus csp22 peptide or csp22^Rsol^ (Felix and Boller, 2003; Figure 3b). To determine whether SlCORE confers responsiveness to csp22^Rsol^ in nonresponsive plants, we used Arabidopsis transgenic lines expressing SlCORE, which have been previously shown to respond to the consensus csp22 peptide (Wang *et al*., 2016). As shown in Figure 5c, Arabidopsis transgenic lines expressing SlCORE gained the ability to respond to csp22^Rsol^, with a similar intensity to that displayed when using consensus csp22 peptide (Figures 5c, S12; Wang *et al*., 2016). Moreover, sustained exposure to csp22^Rsol^ caused a significant inhibition of growth in Arabidopsis seedlings expressing SlCORE, but not in wild‐type plants (Figure 5d). These results indicate that heterologous expression of SlCORE is able to confer responsiveness to csp22^Rsol^ in nonresponsive plants, and SlCORE‐mediated perception of csp22^Rsol^ triggers both early and late immune‐related responses in Arabidopsis.

### Expression of the csp22 receptor SlCORE confers increased resistance to *Ralstonia solanacearum* in Arabidopsis

**Figure 6:**
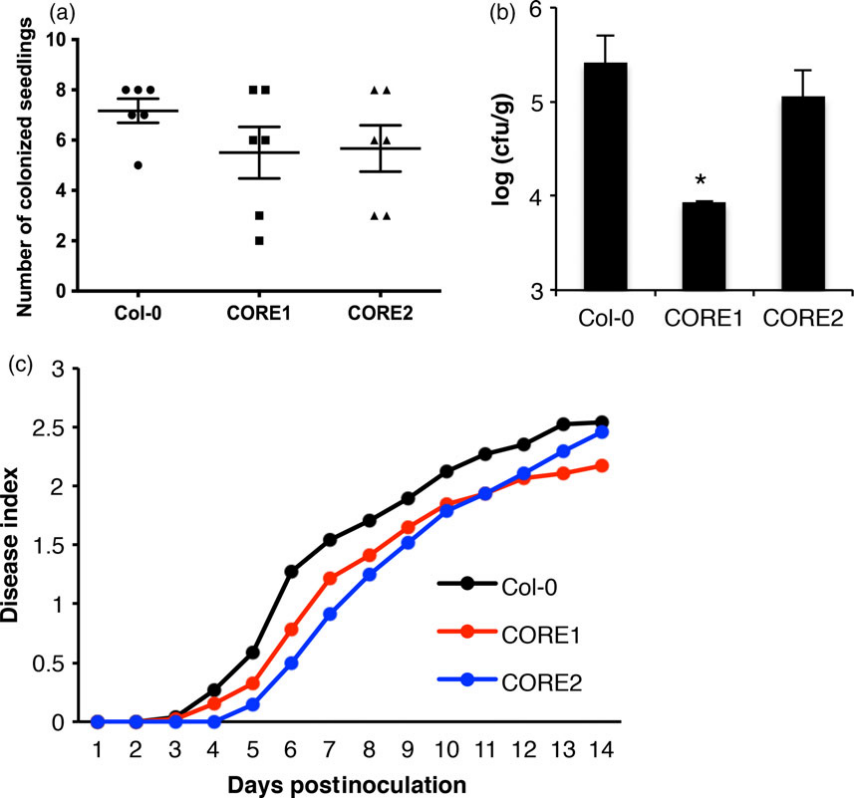
Expression of SlCORE confers increased resistance to *Ralstonia solanacearum* in Arabidopsis. (a and b) A 10^5^ cfu/mL suspension of *R. solanacearum* was used to inoculate roots of 8‐day‐old Arabidopsis seedlings in MS plates. (a) Number of seedlings colonized by *R. solanacearum,* 2 days after root inoculation, in six independent biological replicates (average ± SE;* n* = 6). Eight seedlings were inoculated in each biological replicate. Each symbol represents the number of seedlings showing a detectable number of bacteria in the shoot in each biological replicate. The minimum number of detectable bacteria is 20 cfu. (b) Number of colony‐forming units (cfu) of *R. solanacearum* detected in the shoot 2 days after root inoculation. *N* = 8 for Col‐0; *N* = 3 for the CORE lines (the rest of the samples showed no detectable bacteria). A representation considering all the values (*N* = 8) is shown in the Figure S13. The result is representative of six independent biological replicates. Asterisk indicates significant differences compared to the control according to a Student's *t*‐test (*P* < 0.05). (c) A 10^8^ cfu/mL suspension of *R. solanacearum* was used to soak the pots of 4‐ to 5‐week‐old Arabidopsis plants. Twenty plants per genotype were used. The graph represents the progression of the average wilting symptoms over 14 days. The experiment was performed three times with similar results.

Given that the transgenic expression of SlCORE conferred responsiveness to csp22^Rsol^, we sought to determine whether this csp22^Rsol^ responsiveness has an impact on Arabidopsis resistance to *R. solanacearum*. To this end, we inoculated roots of Arabidopsis seedlings grown in sterile MS medium with a suspension of *R. solanacearum* and recorded the presence of bacteria in shoots 2 days after inoculation. Most wild‐type seedlings were colonized by *R. solanacearum*, showing detectable bacterial numbers in shoots (Figure 6a). However, a larger proportion of Arabidopsis seedlings expressing SlCORE did not contain detectable numbers of *R. solanacearum* colony‐forming units in shoots 2 days after root inoculation, reflecting the inability of bacteria to colonize these plants at this time point (Figure 6a). Moreover, among those seedlings colonized by *R. solanacearum*, shoots of wild‐type seedlings supported higher bacterial loads than seedlings expressing the SlCORE receptor (Figures 6b, S13). Furthermore, mature transgenic plants expressing SlCORE showed a small but reproducible delay in the development of disease symptoms, compared to wild‐type plants, in soil‐drenching infection assays (Figure 6c). These results suggest that transgenic expression of SlCORE confers increased resistance to root infection by *R. solanacearum* in Arabidopsis.

## Discussion

Although the perception of flg22 is a well‐studied example of recognition of a bacterial elicitor by most plant species, certain bacterial strains, such as *Pcal* ES4326 and strains from *A. tumefaciens* or *R. solanacearum*, have developed polymorphisms in the flg22 sequence, which abolish recognition by FLS2 (Clarke *et al*., 2013; Felix *et al*., 1999). This represents an additional level of the well‐accepted arms race between pathogens and plants, and questions the previous idea that microbial elicitors constitute evolutionarily constrained PAMPs, therefore stable targets for recognition by plant cells. Interestingly, specific *Solanaceae* plants have an additional receptor, named FLS3, which can recognize a flagellin peptide different from flg22, named flgII‐28 (Hind *et al*., 2016). This receptor can perceive the flgII‐28 peptide from different *Pseudomonas* strains, including *Pcal* ES4326 (Hind *et al*., 2016), although it was unclear whether it can perceive flgII‐28 from other polymorphic flagellin proteins. Our systematic approach shows that *Solanaceae* plants are unable to perceive synthetic flgII‐28 or full flagellin from *R. solanacearum*. This lack of recognition suggests that flagellin from *R. solanacearum* has undergone a very efficient evolution process to avoid recognition by PRRs from *Solanaceae* plants, while keeping its ability to form a functional flagellum.

The conserved csp22 peptide from the CSP from several bacterial species has been found to elicit responses in specific plant species from the *Solanaceae* family (Felix and Boller, 2003). CSPs constitute a large family of abundant RNA‐binding proteins in Gram‐negative bacteria. Their name reflects the original observation that cold temperature leads to hyperaccumulation of these proteins. However, genes encoding CSP proteins are not only expressed in cold temperatures, but also constitutively and under other stresses (Bae *et al*., 2000). Besides their known roles in the regulation of cellular growth and adaptation to environmental conditions, recent studies have shown that specific CSPs are important for pathogenicity in the enterobacterial pathogen *Salmonella enterica*, where they are induced at higher temperatures, revealing a role for CSPs in bacteria–host interaction (Michaux *et al*., 2017). In *R. solanacearum*, genes encoding proteins that contain csp22‐like sequences are expressed during the infection in tomato (Jacobs *et al*., 2012). According to our model, *R. solanacearum* encounters a stress‐inducing environment when invading plant tissues, which may in turn lead to the hyperaccumulation of CSP proteins. As it has been proposed for other abundant PAMPs present inside bacterial cells (e.g. Ef‐tu; Kunze *et al*., 2004), leakiness of damaged bacteria during the infection process or active export of CSPs may lead to the exposure of csp22. This elicitor could then be perceived at the plant plasma membrane by the extracellular domain of plant PRRs (Saur *et al*., 2016; Wang *et al*., 2016), triggering a signalling cascade that ultimately results in the onset of immunity.

Our work shows that csp22^Rsol^ is able to trigger robust immune responses in tomato, *N. benthamiana,* and tobacco (Figures 3 and 4). Novel elicitation activities found using synthetic peptides may raise concerns regarding potential peptide contamination (Mueller *et al*., 2012b). During the course of this work, we have used other peptide preparations with broad elicitor activities, such as flg22^Pto^ and flgII‐28^Pto^. However, our csp22^Rsol^ peptide solution does not trigger any detectable response in Arabidopsis, ruling out a contamination with flg22^Pto^ (see Figures 1 and 3), while the absence of elicitation in pepper rules out contamination with flgII‐28^Pto^ (see Figures 1 and 3).

Compared to flg22^Pto^, csp22^Rsol^ triggers a weaker ROS burst or MAPK activation at early time points. Interestingly, however, both peptides show a comparable activity when a late response, such as the inhibition of seedling growth, is monitored (Figure 4b and c). Additionally, pretreatment of tomato tissues with csp22^Rsol^ triggers a significant increase in resistance to subsequent *R. solanacearum* infection (Figure 4d), similar to what is usually reported for flg22^Pto^ treatment. This suggests that the outcome of the perception of both peptides is comparable at late time points or after sustained exposure to the elicitor.

Intriguingly, responses to csp22 are reported to be age dependent and are only consistent in leaves from mature plants (older than 5 weeks old; Saur *et al*., 2016; Wang *et al*., 2016). We found a similar pattern for csp22^Rsol^ (Figure 3b). This age dependency is counterintuitive, as it suggests that plants only mount this defence system after a certain age, when the developmental programme of plants is likely to prioritize reproduction rather than immune responses. However, our analysis showed that the plant response to csp22^Rsol^ seems to be more consistent in root tissues. Contrary to the age‐dependent response in leaves, roots of *N. benthamiana* seedlings (7 days old) consistently responded to csp22^Rsol^ (Figure 3c). Furthermore, *N. benthamiana* seedlings displayed a robust reduction in growth after sustained exposure to csp22^Rsol^, showing a remarkable inhibition of root growth (Figure 4c). These results indicate that roots are better equipped for the perception of csp22 compared to leaves at early stages of plant development, and suggest that the biological relevance of CSP perception may be more significant for the perception of csp22 peptides from soil‐borne pathogens, such as *R. solanacearum*. Interestingly, Arabidopsis transgenic plants expressing SlCORE became more resistant to colonization by *R. solanacearum* after root inoculation (Figure 6), supporting the notion that csp22 recognition in roots is able to contribute to disease resistance.

A large number of effectors from different pathogens, including bacterial T3Es, have been found to suppress elicitor‐dependent immune responses (Macho and Zipfel, 2015). Most experimental set‐ups employed to activate elicitor‐triggered immune responses in laboratory conditions involve the treatment with flg22 peptide from *Pseudomonas*, regardless of whether the studied immune‐suppressing microbe (either bacterium, fungus or oomycete) contains an immunogenic flagellin or not. Although this approach has been very helpful in the identification of the immunosuppression capacity of many microbial molecules, it raises the question of whether such microbes are able to suppress immunity activated by their own elicitors. The infection process by *R. solanacearum* most likely involves at least a quantitative suppression of elicitor‐triggered responses, mediated either by T3Es (Mukaihara *et al*., 2016; Sang *et al*., 2018) or other molecules (Tran *et al*., 2016). From a practical point of view, our findings show that it is possible to use an actual *R. solanacearum* elicitor (csp22^Rsol^) to experimentally trigger immunity in *Solanaceae*, instead of employing a heterologous elicitor from *Pseudomonas*, such as flg22.

The csp22 peptide is not perceived outside of the *Solanaceae* family (Felix and Boller, 2003). Accordingly, Arabidopsis plants are unable to perceive csp22^Rsol^, although transgenic Arabidopsis plants expressing SlCORE become responsive to csp22^Rsol^. Interestingly, when we inoculated roots of Arabidopsis seedlings with *R. solanacearum*, we noticed that a considerable proportion of transgenic seedlings expressing SlCORE did not contain any detectable bacteria in the shoot (Figure 6a). Although this hinders the comparison of bacterial loads between wild‐type and SlCORE transgenic plants (creating a bias due to the presence of samples without actual numeric values; see Figures 6b and S13), it also suggests that expression of SlCORE increases resistance to bacterial colonization in Arabidopsis seedlings. This is supported by the observation that transgenic plants expressing SlCORE showed a delay in the development of disease symptoms in soil‐drenching infection assays (Figure 6c). This observation indicates that, despite its ability to suppress immunity, *R. solanacearum* may not be able to cope as efficiently when facing the constitutive expression of additional PRRs components. Similarly, although *R. solanacearum* is able to cause infection in Arabidopsis, which contains the EF‐Tu receptor (EFR), tomato transgenic plants expressing EFR have been reported to become more resistant to *R. solanacearum* (Lacombe *et al*., 2010). Our results indicate that the csp22^Rsol^ peptide is not perceived by several important crops for which *R. solanacearum* is an agricultural threat, both within the *Solanaceae* family (e.g. pepper; Figure 3) and others (e.g. bean and peanut; data not shown). The fact that csp22^Rsol^ is not perceived in these species suggests that transfer of SlCORE, layered atop the pre‐existing defences of a given crop plant or additional PRRs, could significantly contribute to the generation of *R. solanacearum*‐resistant crops.

## Experimental procedures

### Plant material and growth conditions

Except where indicated otherwise, plants used in this study were grown in an environmentally controlled growth room at 22 °C with a 16‐h photoperiod, a 65% humidity and a light‐intensity of 100–150 mE/m^2^/s^1^. Arabidopsis transgenic lines expressing SlCORE (Wang *et al*., 2016) were kindly provided by Georg Felix.

### Chemicals and peptides

All the chemicals used in this study were purchased from Sigma‐Aldrich, St. Louis, MO, USA, unless otherwise stated. Flg22, flgII‐28 and csp22 peptides were purchased from ABclonal, using the following amino acid sequences: Flg22^Pto^: TRLSSGLKINSAKDDAAGLQIA; flg22^Rsol^: QRLSTGLRVNSAQDDSAAYAAS; flgII‐28^Pto^: ESTNILQRMRELAVQSRNDSNSSTDRDA; flgII‐28^Rsol^: QVENNLQRMRQLAVESNNGGLSAADQTN; csp22^Rsol^: ATGTVKWFNETKGFGFITPDGG; csp22 (consensus): AVGTVKWFNAEKGFGFITPDDG.

### Agrobacterium‐mediated transient expression

Agrobacterium‐mediated transient expression in *N. benthamiana* was performed as described (Li, 2011). Before infiltration, the bacterial suspension was adjusted to a final OD^600^ of 0.5. Samples were taken at 1–3 days postinoculation (dpi) for analysis based on experimental requirements, as indicated for each specific experiment.

### Measurement of PAMP‐triggered ROS burst

Reactive oxygen species (ROS) production in leaves upon PAMP treatment was measured following the protocol described by Sang and Macho (Sang and Macho, 2017). *Nicotiana benthamiana, Nicotiana tabacum* (cv. Petit Havana)*,* eggplant (Zebrina), pepper (California Wonder) and tomato (Moneymaker) plants were grown on soil. Leaf discs were taken from 3‐ to 6‐week‐old plants (as indicated for each specific experiment) for ROS measurement. Four‐ to five‐week‐old Arabidopsis plants grown in short day conditions were used.

The measurement of ROS production in roots was similar except for several minor modifications in sample collection. Tomato seeds were germinated on filter paper with water in a Petri dish, and 7‐day‐old seedlings were used for ROS assay. *Nicotiana benthamiana* and Arabidopsis seeds were first germinated on 1/2 MS (Murashige‐Skoog) solid medium for 7 days and then transferred to 1/2 MS liquid culture for 4 days before ROS measurement. Roots of seedlings were cut into one‐centimetre‐long sections and allowed to recover for 5 h in 96‐well plates with 100 μL H_2_O in each well. Eight root sections were analysed for each sample, using 100 nm of the corresponding peptide. For root assays, luminol was replaced by the more sensitive derivative L‐012 (Wako Chemical, Japan), as stated in the figure legends. It is worth noticing that approximately 20% of tomato root samples showed very weak or no response to elicitor treatment, and this is most likely due to the sensitivity of tomato roots to the handling process. Although this leads to higher variation in ROS results, it did not affect the conclusions of the assays.

### MAPK activation assay

To measure MAPK activation in leaves, intact leaves were immersed in 1 μm elicitor solution in a beaker and vacuum‐infiltrated for 3 min. After 15 min, four leaf discs with 8 mm diameter were collected and frozen in liquid nitrogen for further analysis. Seedlings were immersed in 1 μm elicitor solution directly for 10/15 min and then were collected and frozen immediately in liquid nitrogen for further analysis. Western blots to determine MAPK activation were performed as described (Macho *et al*., 2012). Blots were incubated with anti‐actin (Agrisera AS132640) to verify equal loading.

### Protein expression and purification

The FliC gene from *Pseudomonas syringae* DC3000 or *R. solanacearum* GMI1000 and GFP gene were cloned into PET13‐strep‐msb‐his vector and then transformed to Rosetta *E. coli* cells to express the His‐MSB‐strep tag fused protein. Three columns were sequentially used for protein purification: Ni column (Merck, Darmstadt, Germany), anion‐exchange column (GE Healthcare, Pittsburgh, PA) and strep column (GE Healthcare, Chicago, IL), following the procedures described by the manufacturers. The genes were cloned into pDest566 (Addgene, Cambridge, MA) to generate His‐MBP tag fusion proteins. Ni column (Merck, Darmstadt, Germany), desalt column (GE Healthcare, Chicago, IL) and MBP column (NEB, Ipswich, MA) were used sequentially to purify the resulting proteins. Gel filtration (GE Healthcare, Chicago, IL) was used finally to remove unspecific proteins using Äkta purifier (GE Healthcare, Chicago, IL).

### Protein identification by LC‐MS/MS

*Escherichia coli* protein extracts were digested with trypsin and analysed by LC‐MS/MS following the protocol described by Sang *et al*. (2018). The mass spectra were submitted to Mascot Server (version 2.5.1, Matrix Science, London, UK) for peptide identification and searched against the *E. coli* protein database supplemented with GFP protein sequence and flagellin protein sequences from *P. syringae* and *R. solanacerum*.

### Seedling growth inhibition

Arabidopsis *seeds* were sterilized for 10 min in 75% ethanol and 0.1% Triton X‐100, then washed two times in 75% ethanol and dried in 100% ethanol. Seed were germinated on 1.5% agar plates with MS medium for 48 h before transfer to 48‐well plates containing 500 μL of liquid MS or liquid MS containing peptides (100 nm flg22 or 1 μm csp22). Seedlings grew at 21 °C with 12 h of light and 12 h of darkness. Fresh weight was recorded for each seedling 9 days after transfer to liquid media. *Nicotiana benthamiana* seeds were sterilized, sown and weighted as described above, but transferred to 24‐well plates with 3 mL of liquid MS or liquid MS containing peptides.

### *Ralstonia solanacearum* infection assays

*Ralstonia solanacearum* GMI1000 was grown overnight in B liquid medium (Plener *et al*., 2012). For soil inoculations, 20 four‐week‐old *A. thaliana* plants per genotype were grown in Jiffy pots (Jiffy International, Kristiansand, Norway) and inoculated by soil drenching with a 5 × 10^7^ colony‐forming units/mL bacterial suspension. One litre of inoculum was used to soak 20 *A. thaliana*‐containing pots. After 20‐min incubation, plants were removed from the bacterial solution and placed on a bed of potting mixture soil in a new tray. Visual scoring of disease symptoms according to a scale ranging from ‘0’ (no symptoms) to ‘4’ (complete wilting) was performed as described previously (Vailleau *et al*., 2007). For internal growth curve assays (IGC), 8‐day‐old Arabidopsis seedlings were grown on MS plates and inoculated with 10^5^ cfu/mL GMI1000 bacterial suspension, as described in Lu *et al*. (2018), with minor modifications: IGC was performed by collecting aerial parts of Arabidopsis seedlings at 2 dpi, followed by washing in sterile water. Colony‐forming units were counted by spreading serial dilutions on solid B medium. An unpaired Student's *t* test was used to determine statistical differences between the samples. In cases where the data did not adjust to a normal distribution, a nonparametric Mann–Whitney *U* test was used.

For induced resistance assays in tomato, leaves of 4‐week‐old tomato plants (cultivar M82) were infiltrated with 1 μm csp22^Rsol^ or water (mock). Twenty‐four hours later, the same leaves were infiltrated with 10^6^ cfu/mL GMI1000 bacterial suspension. Three dpi, three 8‐mm leaf discs were excised using a cork‐borer, weighed and ground in 10 mm MgCl_2_. Colony‐forming units were counted by spreading serial dilutions on solid B medium. An unpaired Student's *t*‐test was used to determine statistical differences between the samples.

## Conflict of interest

The authors declare no conflict of interest.

## Supporting information


**Figure S1** Sequence alignment of the amino acid sequence of FliC from different bacterial species. The major elicitor peptides flg22 and flgII‐28 are highlighted. Pto, *Pseudomonas syringae*; Rs, *Ralstonia solanacearum*.
**Figure S2** Flg22 or flgII‐28 from *Ralstonia solanacearum* do not elicit responses in leaves of *Solanaceae* plants. Dynamics of oxidative burst from the assays described in the Figure 1a, measured in a luminol‐based assay as relative luminescence units (RLU) during 60 min. Values are average ± SE (*n* = 8).
**Figure S3** Flg22 or flgII‐28 from *Ralstonia solanacearum* do not elicit responses in roots of *Solanaceae* plants. Dynamics of oxidative burst from the assays described in the Figure 1b, measured in a luminol‐based assay as relative luminescence units (RLU) during 60 min. Values are average ± SE (*n* = 8).
**Figure S4** Flg22 or flgII‐28 from *Ralstonia solanacearum* do not elicit MAPK activation in leaves of *Solanaceae* plants. MAPK activation assay in leaves of 5‐week‐old tomato (a), *Nicotiana benthamiana* (b) or Arabidopsis plants after treatment with 1 μm of the indicated peptides or water (mock) for 10, 15, or 20 min as indicated. Immunoblots were analysed using antiphosphorylated MAPK antibody (α‐pMAPK). Immunoblots were also analysed using α‐actin antibody to verify protein accumulation. Molecular weight (kDa) marker bands are indicated for reference.
**Figure S5** Schematic representation of the 6His‐MSB‐FliC‐Strep recombinant protein.
**Figure S6** Purified recombinant flagellin from *Ralstonia solanacearum* does not elicit responses in leaves of *Solanaceae* plants. Dynamics of oxidative burst from the assays described in the Figure 2a, measured in a luminol‐based assay as relative luminescence units (RLU) during 60 min. Values are average ± SE (*n* = 8).
**Figure S7** Purified recombinant flagellin from *Ralstonia solanacearum* does not elicit responses in roots of *Solanaceae* plants. Dynamics of oxidative burst from the assays described in the Figure 2b, measured in a luminol‐based assay as relative luminescence units (RLU) during 60 min. Values are average ± SE (*n* = 8).
**Figure S8** His‐MBP‐FliC purification and quality control. (a) Schematic representation of the His‐MBP‐FliC recombinant protein. (b) Representative gel filtration analysis of the recombinant proteins. A red box indicates the fractions that were pulled together and used for elicitation assays. (c) SDS‐PAGE analysis of the final purified protein used for elicitation assays. (d) LC‐MS/MS analysis after tryptic digestion of the final purified FliC_Rs used for elicitation assays. Highlighted sequences represent the detected peptides.
**Figure S9** Purified recombinant His‐MBP‐FliC from *Ralstonia solanacearum* does not elicit responses in leaves of *Solanaceae* plants. Oxidative burst triggered by 100 nm of the indicated recombinant proteins in tomato (a,b), *Nicotiana benthamiana* (c,d), and Arabidopsis (e,f), measured in a luminol‐based assay as relative luminescence units (RLU) during 60 min. b,d and f show the dynamics of oxidative burst from the assays shown in a,c and e, respectively. Values are average ± SE (*n* = 8). The experiments were repeated at least three times with similar results.
**Figure S10** Amino acid sequence alignment of csp22‐like peptides from *Ralstonia solanacearum* GMI1000 proteins. Locus identifiers for the genes encoding these proteins are shown as RSpXXX or RScXXX, together with their annotation. Csp22_Rsol indicates the peptide used in this work (sequence from Cspd3). Csp22_Consensus indicates the consensus peptide characterized in a previous report (Wang *et al*., 2016).
**Figure S11** Csp22 from *Ralstonia solanacearum* is perceived in leaves of specific *Solanaceae* plants. Dynamics of oxidative burst from the assays described in the Figure 3a, measured in a luminol‐based assay as relative luminescence units (RLU) during 60 min. Values are average ± SE (*n* = 8).
**Figure S12** The CORE receptor confers responsiveness to csp22 from *Ralstonia solanacearum*. (a and b) Dynamics of oxidative burst from the assays described in the Figure 5a and b, measured in a luminol‐based assay as relative luminescence units (RLU) during 60 min. (c) Accumulation of SlCORE‐GFP in Arabidopsis transgenic plants. The immunoblot was analysed using anti‐GFP antibody (α‐GFP). A molecular weight (KDa) marker band is indicated for reference, and a nonspecific band (marked with an asterisk) is used as control for equal loading. (d) Total RLU accumulated during 60 min from the assay described in the Figure 5c. Values are average ± SE (*n* = 8).
**Figure S13** Expression of SlCORE significantly reduce *Ralstonia solanacearum* infection in Arabidopsis. Number of colony‐forming units (cfu) of *R. solanacearum* detected in the shoot 2 days after root inoculation. These data correspond to the experiment shown in the Figure 6b. In this representation, samples where no bacteria were detected are represented as 20 cfu, which corresponds to the minimum number of detectable bacteria. Therefore, *N* = 8 for all genotypes. The result is representative of six independent biological replicates. Asterisks indicate significant differences compared to the control according to a Student's *t*‐test (*P < 0.05; ***P < 0.001).
