# Supplementary material for: The Ralstonia solanacearum csp22 peptide, but not flagellin‐derived peptides, is perceived by plants from the Solanaceae family
Source: Plant Biotechnol J. 2018 Jan 22;16(7):1349–62. doi: 10.1111/pbi.12874 (PMC5999195; doi:10.1111/pbi.12874)

Figure S1

```
                                                   flg22
Pto_DC3000    1 MALTVNTNVASLNVQKNLGRASDALSTSMTRLSSGLKINSAKDDAAGLQIATKITSQIRGQTMAIK
Pto_T1        1 MALTVNTNVASLNVQKNLGRASDALSTSMTRLSSGLKINSAKDDAAGLQIATKITSQIRGQTMAIK
Rs_GMI1000    1 MSLSLNTNISSLQTQQALSQSQSALQKSLQRLSTGLRVNSAQDDSAAYAASSSLTTTLNSQTQGIQ

Pto_DC3000   67 NANDGMSLAQTAEGALQESTNILQRMRELAVQSRNDSNSATDREALNKEFTAMSSELTRIAQSTNL
Pto_T1       67 NANDGMSLAQTAEGALQESTNILQRMRELAVQSRNDSNSSTDRDALNKEFTAMSSELTRIAQSTNL
Rs_GMI1000   67 NANGANSYLQTADSYLGQVENNLQRMRQLAVESNNGGLSAADQTNLDKEYQQLATANKNIETNANY
                           flgII-28

Pto_DC3000  133 NGKNLLDGSASTMTFQVGSNSGASNQISLTLSASFDANTLGVGSAISITGADSATSEAAFSAAVAA
Pto_T1      133 NGKNLLDGSASTMTFQVGSNSGSSNQITLTLSASFDANTLGVGSAVTIAGSDSTTSETNFSAAIAA
Rs_GMI1000  133 NGNKLFDGSVASTTFQYGQNAAT....DVTTVTNVNMSTFG.....TLTG.TSVTSAANATAAQAA

Pto_DC3000  199 IDSALQTINSTRADLGAAQNRLTSTISNLQNINENASAALGRVQDTDFAAETAQLTKQQTLQQAST
Pto_T1      199 IDSALQTINSTRADLGAAQNRLTSTISNLQNINENASAALGRVQDTDFAAETAQLTKQQTLQQAST
Rs_GMI1000  189 IDTDLTSLKAARASLGAQQSGLASTINTLTSNNTALSAAKSTLIDTDYASETSNMTRQNILQQAGT

Pto_DC3000  265 SVLAQANQLPSAVLKLLQ
Pto_T1      265 SVLAQANQLPSAVLKLLQ
Rs_GMI1000  255 AMLAQANSAPNSILNLLKG
```

Figure S2

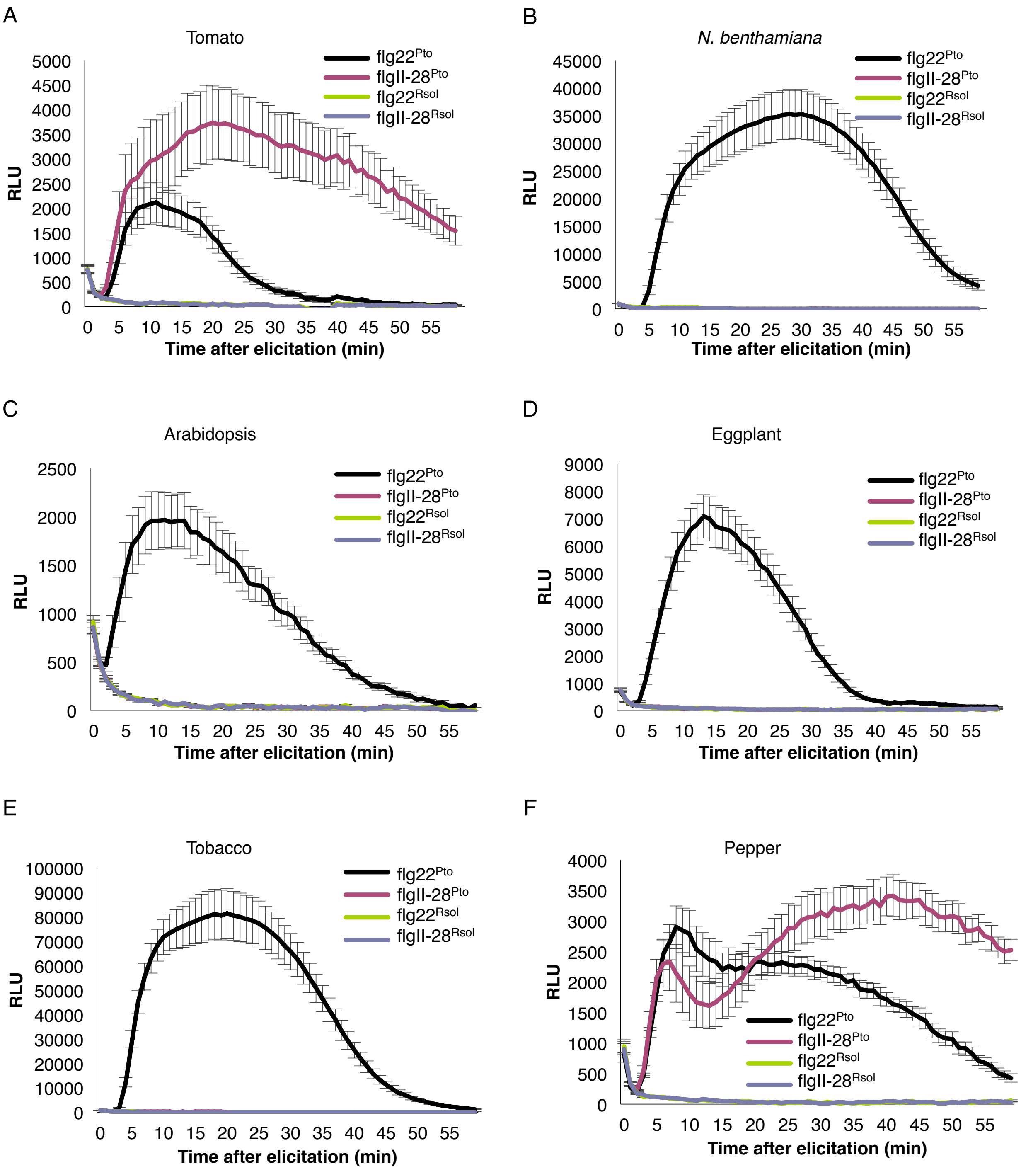
A
Tomato
RLU
5000
4500
4000
3500
3000
2500
2000
1500
1000
500
0
0 5 10 15 20 25 30 35 40 45 50 55
Time after elicitation (min)
flg22^{Pto}
flgII-28^{Pto}
flg22^{Rsol}
flgII-28^{Rsol}
B
N. benthamiana
RLU
45000
40000
35000
30000
25000
20000
15000
10000
5000
0
0 5 10 15 20 25 30 35 40 45 50 55
Time after elicitation (min)
flg22^{Pto}
flgII-28^{Pto}
flg22^{Rsol}
flgII-28^{Rsol}
C
Arabidopsis
RLU
2500
2000
1500
1000
500
0
0 5 10 15 20 25 30 35 40 45 50 55
Time after elicitation (min)
flg22^{Pto}
flgII-28^{Pto}
flg22^{Rsol}
flgII-28^{Rsol}
D
Eggplant
RLU
9000
8000
7000
6000
5000
4000
3000
2000
1000
0
0 5 10 15 20 25 30 35 40 45 50 55
Time after elicitation (min)
flg22^{Pto}
flgII-28^{Pto}
flg22^{Rsol}
flgII-28^{Rsol}
E
Tobacco
RLU
100000
90000
80000
70000
60000
50000
40000
30000
20000
10000
0
0 5 10 15 20 25 30 35 40 45 50 55
Time after elicitation (min)
flg22^{Pto}
flgII-28^{Pto}
flg22^{Rsol}
flgII-28^{Rsol}
F
Pepper
RLU
4000
3500
3000
2500
2000
1500
1000
500
0
0 5 10 15 20 25 30 35 40 45 50 55
Time after elicitation (min)
flg22^{Pto}
flgII-28^{Pto}
flg22^{Rsol}
flgII-28^{Rsol}

Figure S3

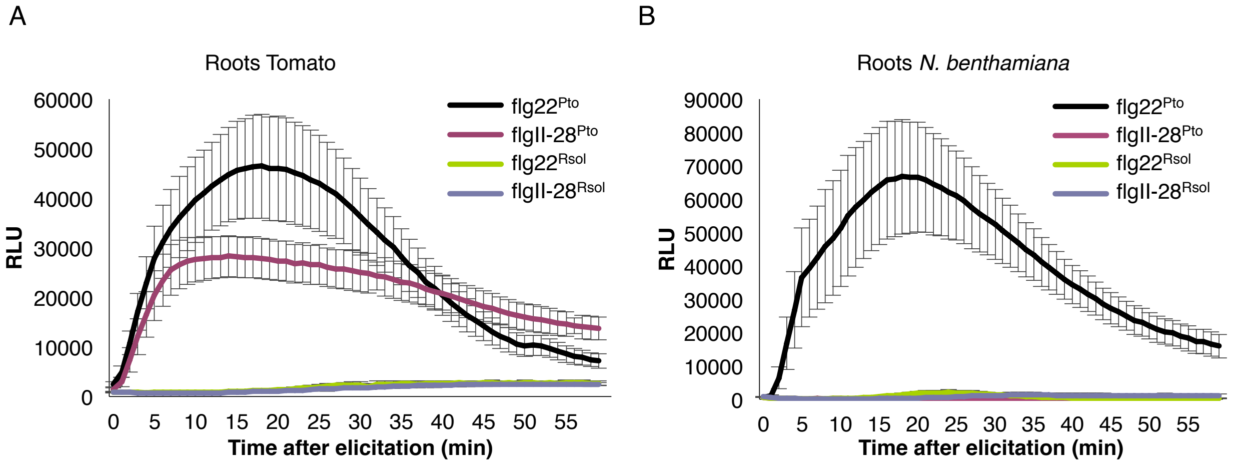
A
B
Roots Tomato
Roots N. benthamiana
RLU
Time after elicitation (min)
flg22Pto
flgII-28Pto
flg22Rsol
flgII-28Rsol
60000
50000
40000
30000
20000
10000
90000
80000
70000
0
5
15
25
35
45
55

Figure S4

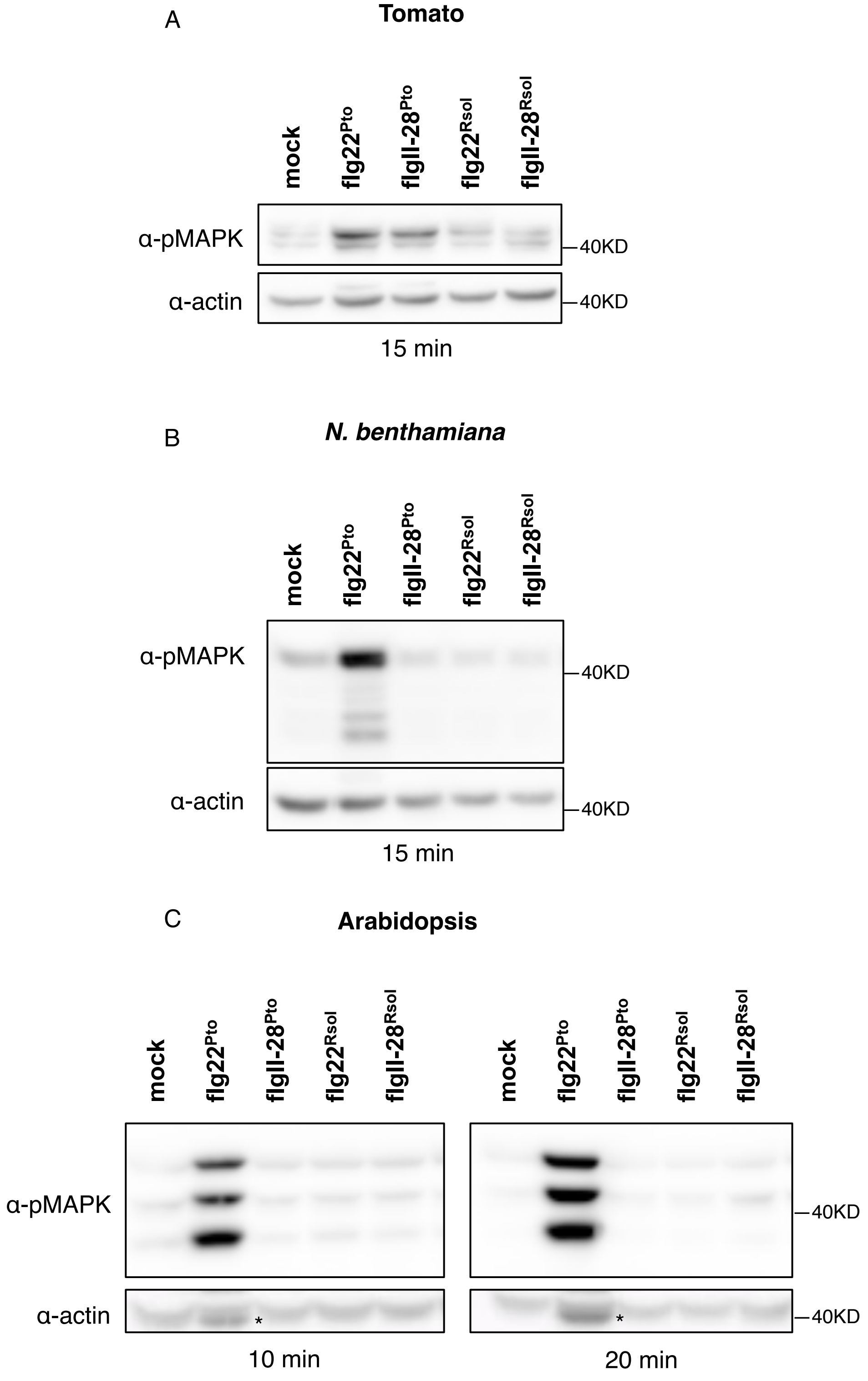
A
Tomato
mock
flg22Pto
flgII-28Pto
flg22Rsol
flgII-28Rsol
α-pMAPK
α-actin
40KD
40KD
15 min
B
N. benthamiana
mock
flg22Pto
flgII-28Pto
flg22Rsol
flgII-28Rsol
α-pMAPK
α-actin
40KD
40KD
15 min
C
Arabidopsis
mock
flg22Pto
flgII-28Pto
flg22Rsol
flgII-28Rsol
mock
flg22Pto
flgII-28Pto
flg22Rsol
flgII-28Rsol
α-pMAPK
α-actin
*
*
40KD
40KD
10 min
20 min

Figure S5

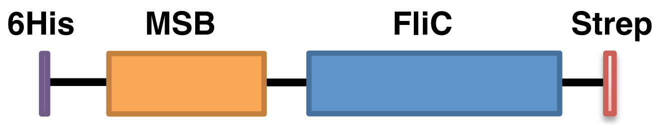

Figure S6

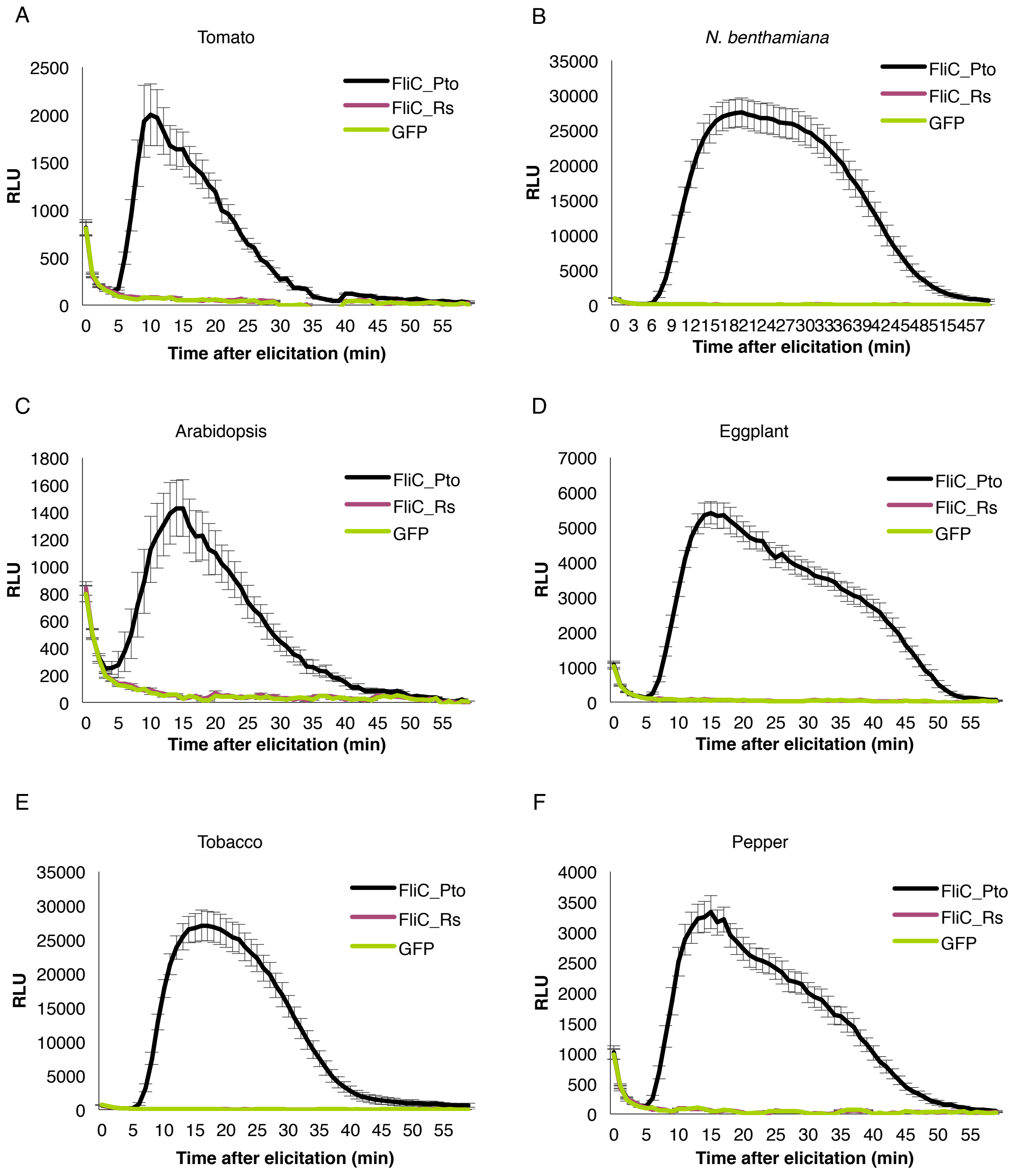
A
Tomato
2500
2000
1500
1000
500
0
RLU
0 5 10 15 20 25 30 35 40 45 50 55
Time after elicitation (min)
FliC_Pto
FliC_Rs
GFP
B
N. benthamiana
35000
30000
25000
20000
15000
10000
5000
0
RLU
0 3 6 9 121518212427303336394245485154 57
Time after elicitation (min)
FliC_Pto
FliC_Rs
GFP
C
Arabidopsis
1800
1600
1400
1200
1000
800
600
400
200
0
RLU
0 5 10 15 20 25 30 35 40 45 50 55
Time after elicitation (min)
FliC_Pto
FliC_Rs
GFP
D
Eggplant
7000
6000
5000
4000
3000
2000
1000
0
RLU
0 5 10 15 20 25 30 35 40 45 50 55
Time after elicitation (min)
FliC_Pto
FliC_Rs
GFP
E
Tobacco
35000
30000
25000
20000
15000
10000
5000
0
RLU
0 5 10 15 20 25 30 35 40 45 50 55
Time after elicitation (min)
FliC_Pto
FliC_Rs
GFP
F
Pepper
4000
3500
3000
2500
2000
1500
1000
500
0
RLU
0 5 10 15 20 25 30 35 40 45 50 55
Time after elicitation (min)
FliC_Pto
FliC_Rs
GFP

Figure S7

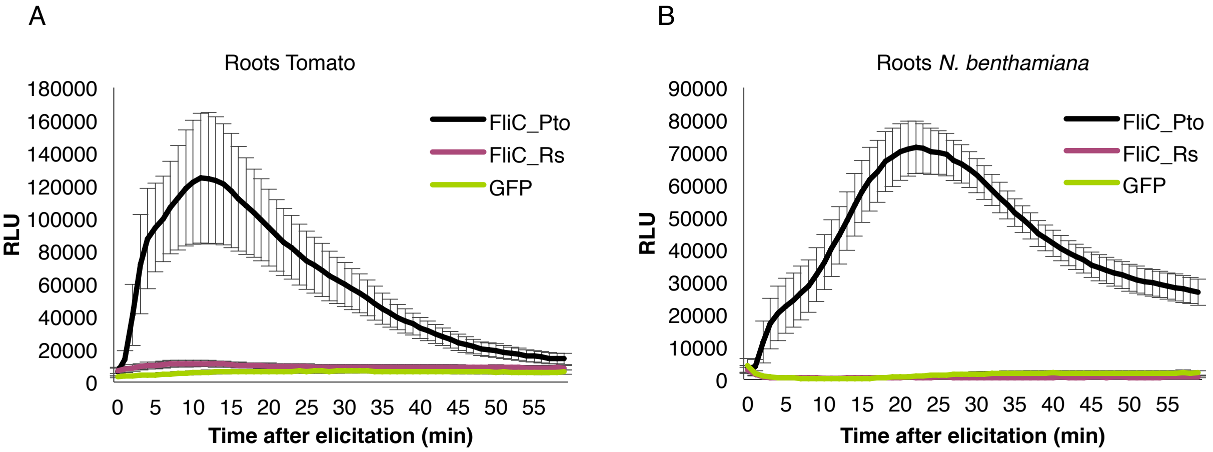
A
Roots Tomato
RLU
180000
160000
140000
120000
100000
80000
60000
40000
20000
0
0 5 10 15 20 25 30 35 40 45 50 55
Time after elicitation (min)
FliC_Pto
FliC_Rs
GFP
B
Roots *N. benthamiana*
RLU
90000
80000
70000
60000
50000
40000
30000
20000
10000
0
0 5 10 15 20 25 30 35 40 45 50 55
Time after elicitation (min)
FliC_Pto
FliC_Rs
GFP

Figure S8

A

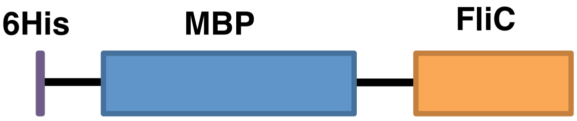

B

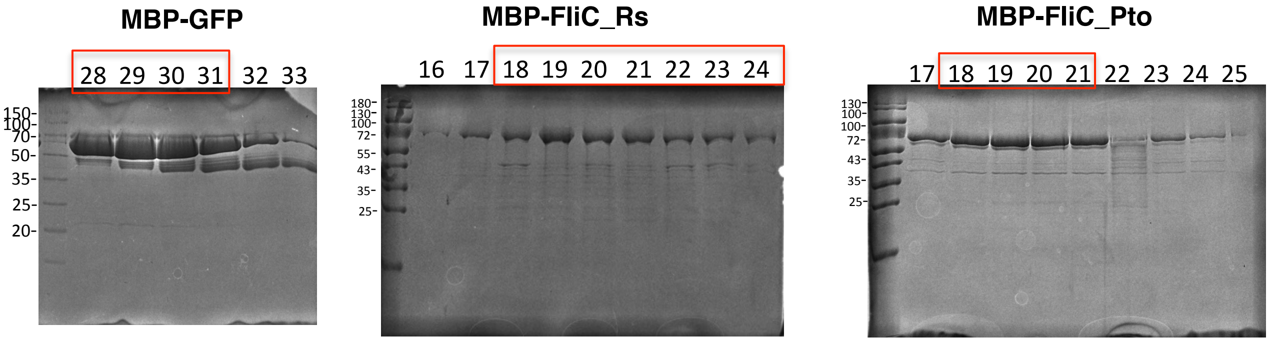

C

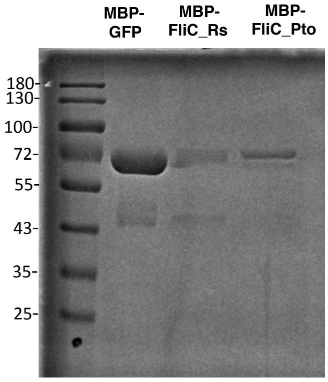

D

Rs (100%), 28,374.5 Da
Rs
12 exclusive unique peptides, 40 exclusive unique spectra, 332 total spectra, 197/273 amino acids (72% cover[illegible]

```
MSLSLNTNIS SLQTQQALSQ SQSALQKSLQ RLSTGLRVNS AQDDSAAYAA
SSSLTTTLNS QTQGIQNANG ANSYLQTADS YLGQVENNLQ RMRQLAVESN
NGGLSAADQT NLDKEYQQLA TANKNIETNA NYNGNKLFDG SVASTTFQYG
QNAATDVTTV TNVNMSTFGT LTGTSVTSAA NATAAQAAID TDLTSLKAAR
ASLGAQQSGL ASTINTLTSN NTALSAAKST LIDTDYASET SNMTRQNILQ
QAGTAMLAQA NSAPNSILNL LKG
```

Figure S9

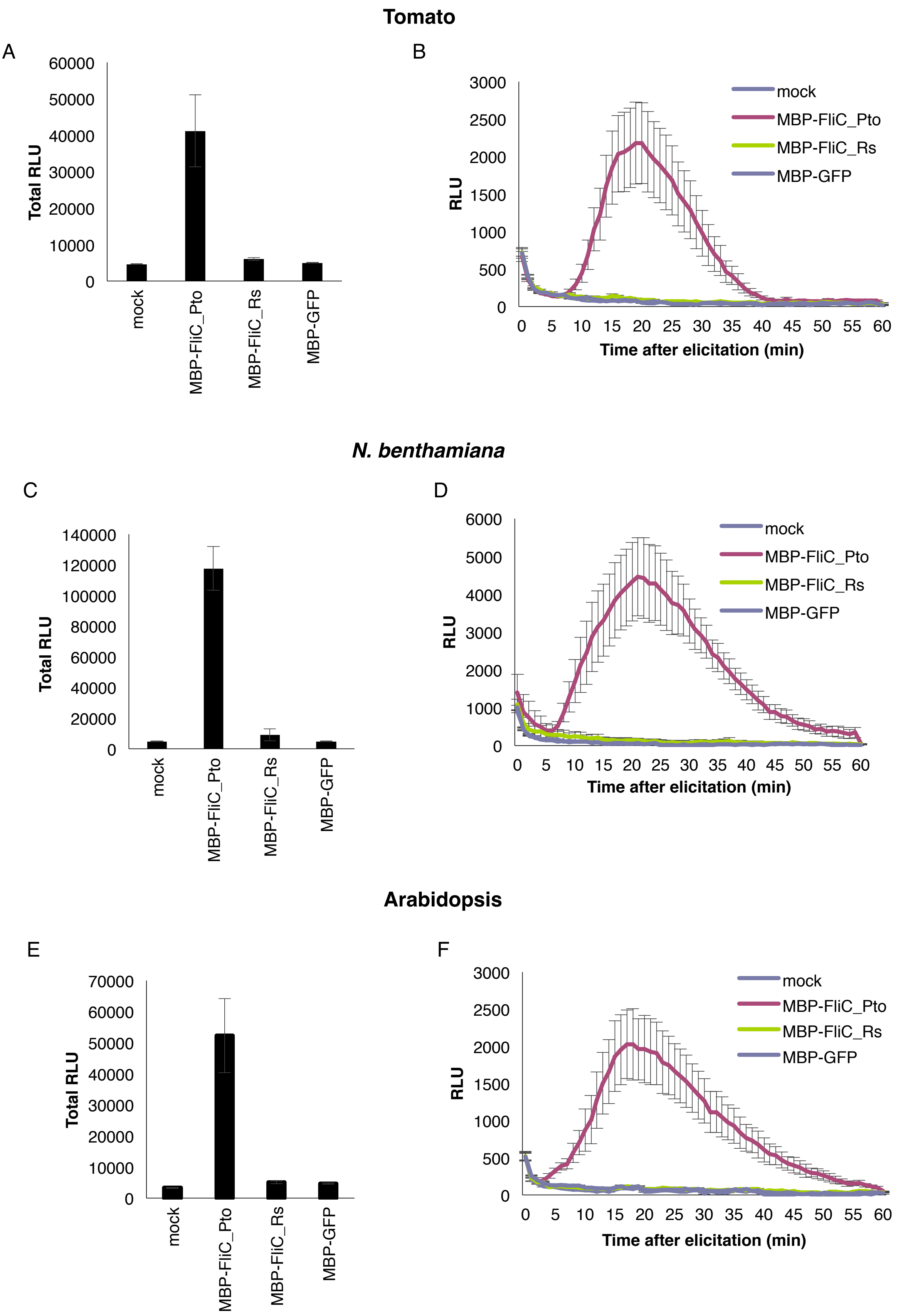

Tomato
A
Total RLU
60000
50000
40000
30000
20000
10000
0
mock
MBP-FliC_Pto
MBP-FliC_Rs
MBP-GFP
B
RLU
3000
2500
2000
1500
1000
500
0
0 5 10 15 20 25 30 35 40 45 50 55 60
Time after elicitation (min)
mock
MBP-FliC_Pto
MBP-FliC_Rs
MBP-GFP
N. benthamiana
C
Total RLU
140000
120000
100000
80000
60000
40000
20000
0
mock
MBP-FliC_Pto
MBP-FliC_Rs
MBP-GFP
D
RLU
6000
5000
4000
3000
2000
1000
0
0 5 10 15 20 25 30 35 40 45 50 55 60
Time after elicitation (min)
mock
MBP-FliC_Pto
MBP-FliC_Rs
MBP-GFP
Arabidopsis
E
Total RLU
70000
60000
50000
40000
30000
20000
10000
0
mock
MBP-FliC_Pto
MBP-FliC_Rs
MBP-GFP
F
RLU
3000
2500
2000
1500
1000
500
0
0 5 10 15 20 25 30 35 40 45 50 55 60
Time after elicitation (min)
mock
MBP-FliC_Pto
MBP-FliC_Rs
MBP-GFP

Figure S10

csp22-like sequences in *Ralstonia solanacearum* GMI1000

```
RSp0002_cspd3    1 ATGTVKWFNETKGFGFITPDGG
RSp1053_cspd2    1 ATGTVKWFNDAKGFGFITPDEG
RSc3156_cspc     1 ETGTVKWFNESKGFGFITPDAG
RSc2466_cspd     1 ANGTVKWFNDAKGFGFISPDEG
csp22_Rsol       1 ATGTVKWFNETKGFGFITPDGG
csp22_Consensus  1 AVGTVKWFNAEKGFGFITPDDG
```

Figure S11

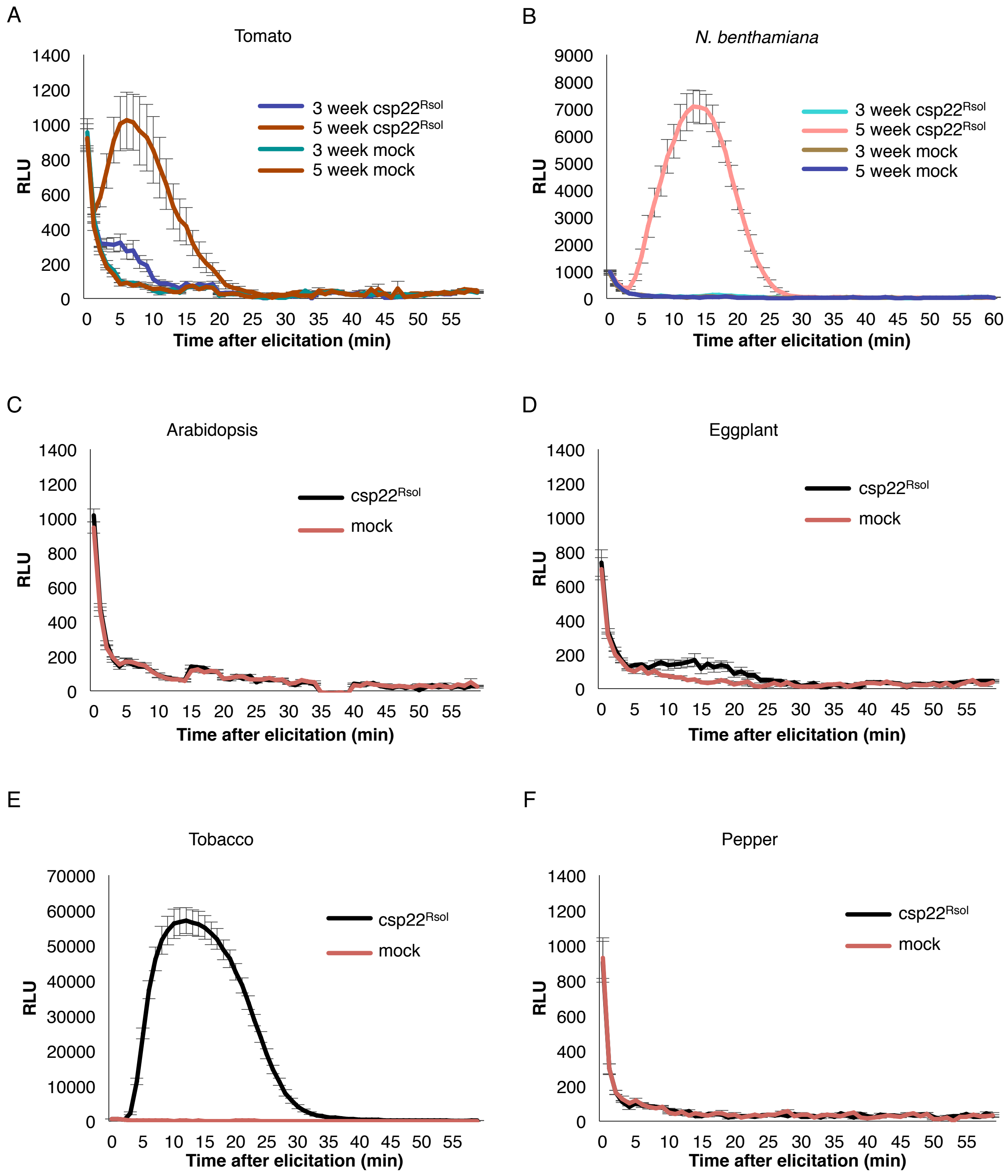
A
Tomato
RLU
Time after elicitation (min)
3 week csp22Rsol
5 week csp22Rsol
3 week mock
5 week mock
B
N. benthamiana
RLU
Time after elicitation (min)
3 week csp22Rsol
5 week csp22Rsol
3 week mock
5 week mock
C
Arabidopsis
RLU
Time after elicitation (min)
csp22Rsol
mock
D
Eggplant
RLU
Time after elicitation (min)
csp22Rsol
mock
E
Tobacco
RLU
Time after elicitation (min)
csp22Rsol
mock
F
Pepper
RLU
Time after elicitation (min)
csp22Rsol
mock

Figure S12

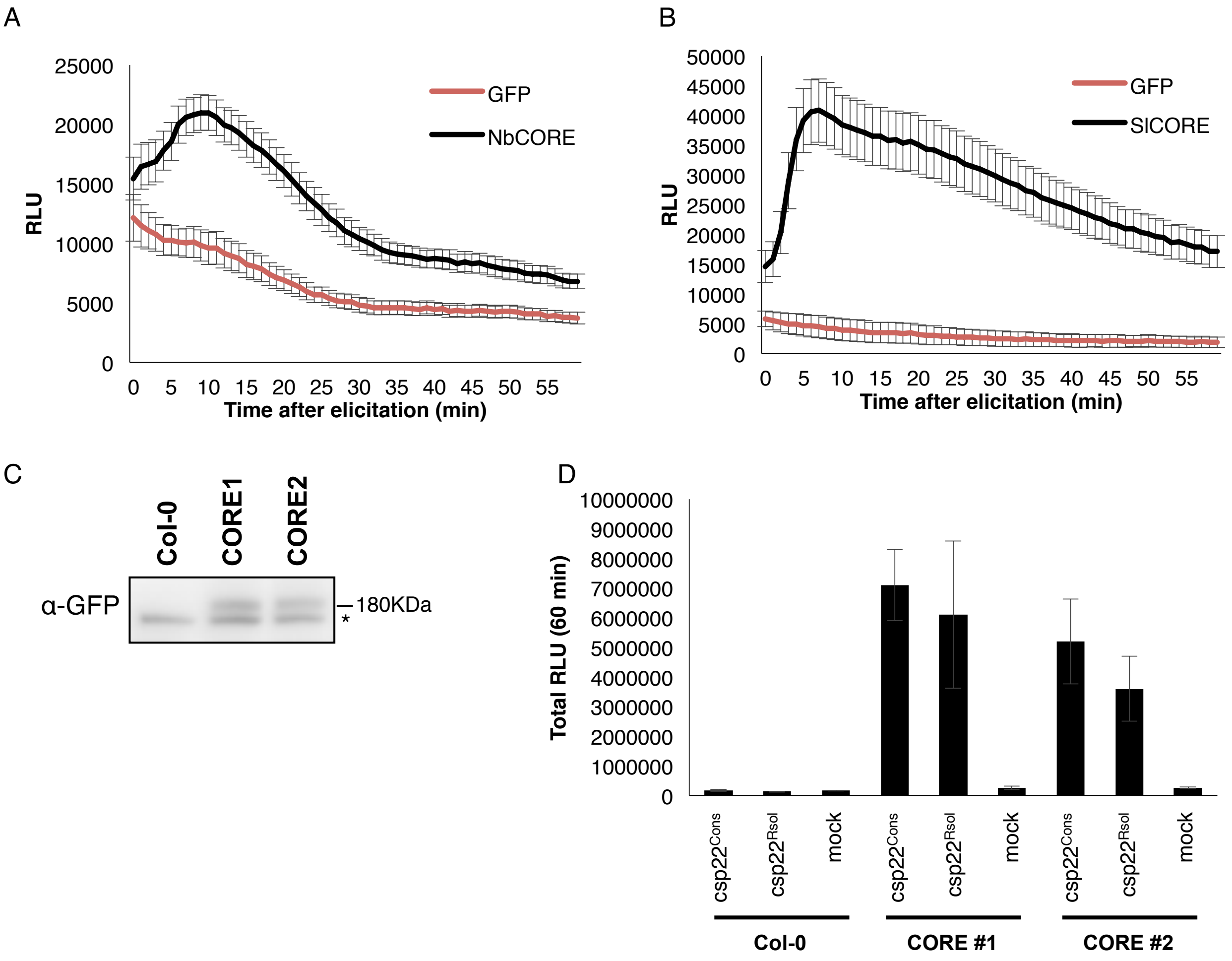
A
25000
20000
15000
10000
5000
0
RLU
GFP
NbCORE
0 5 10 15 20 25 30 35 40 45 50 55
Time after elicitation (min)
B
50000
45000
40000
35000
30000
25000
20000
15000
10000
5000
0
RLU
GFP
SlCORE
0 5 10 15 20 25 30 35 40 45 50 55
Time after elicitation (min)
C
Col-0
CORE1
CORE2
α-GFP
180KDa
*
D
10000000
9000000
8000000
7000000
6000000
5000000
4000000
3000000
2000000
1000000
0
Total RLU (60 min)
csp22Cons
csp22Rsol
mock
Col-0
CORE #1
CORE #2

Figure S13

A

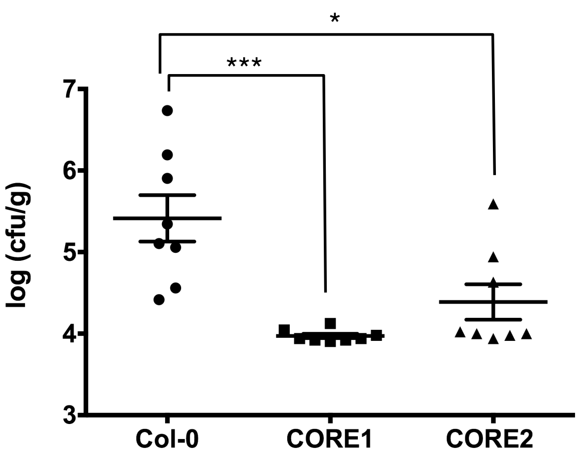

Supplement: Supplementary file 1 — Figure S1 Sequence alignment of the amino acid sequence of FliC from different bacterial species. The major elicitor peptides flg22 and flgII‐28 are highlighted. Pto, Pseudomonas syringae; Rs, Ralstonia solanacearum. Figure S2 Flg22 or flgII‐28 from Ralstonia solanacearum do not elicit responses in leaves of Solanaceae plants. Dynamics of oxidative burst from the assays described in the Figure 1a, measured in a luminol‐based assay as relative luminescence units (RLU) during 60 min. Values are average ± SE (n = 8). Figure S3 Flg22 or flgII‐28 from Ralstonia solanacearum do not elicit responses in roots of Solanaceae plants. Dynamics of oxidative burst from the assays described in the Figure 1b, measured in a luminol‐based assay as relative luminescence units (RLU) during 60 min. Values are average ± SE (n = 8). Figure S4 Flg22 or flgII‐28 from Ralstonia solanacearum do not elicit MAPK activation in leaves of Solanaceae plants. MAPK activation assay in leaves of 5‐week‐old tomato (a), Nicotiana benthamiana (b) or Arabidopsis plants after treatment with 1 μm of the indicated peptides or water (mock) for 10, 15, or 20 min as indicated. Immunoblots were analysed using antiphosphorylated MAPK antibody (α‐pMAPK). Immunoblots were also analysed using α‐actin antibody to verify protein accumulation. Molecular weight (kDa) marker bands are indicated for reference. Figure S5 Schematic representation of the 6His‐MSB‐FliC‐Strep recombinant protein. Figure S6 Purified recombinant flagellin from Ralstonia solanacearum does not elicit responses in leaves of Solanaceae plants. Dynamics of oxidative burst from the assays described in the Figure 2a, measured in a luminol‐based assay as relative luminescence units (RLU) during 60 min. Values are average ± SE (n = 8). Figure S7 Purified recombinant flagellin from Ralstonia solanacearum does not elicit responses in roots of Solanaceae plants. Dynamics of oxidative burst from the assays described in the Figure 2b, measured in a luminol‐ba [file PBI-16-1349-s001.pdf]
